# Cutaneous manifestations of COVID‐19: diagnosis and management

**DOI:** 10.5694/mja2.51621

**Published:** 2022-06-22

**Authors:** Nicole Seebacher, Julie Kirkham, Saxon D Smith

**Affiliations:** ^1^ University of Oxford Oxford UK; ^2^ St James’s Hospital Dublin Ireland; ^3^ Australian National University Canberra ACT

**Keywords:** COVID‐19, Dermatology, Skin diseases, infectious

## Case report

A 48‐year‐old female health care worker of European descent, who was otherwise well and on no regular medications, developed cough symptoms the day before testing positive for coronavirus disease 2019 (COVID‐19). Five days after after symptom onset, she developed rhinorrhoea followed by loss of taste and smell (anosmia and ageusia). On day 7, she developed headaches, palpations, subjective fevers and an eruption on the dorsum of her hands; on day 8, the eruption became pruritic and had spread to her elbows, the dorsum of her feet, and chest (Box 1). The pruritus was successfully treated with an oral antihistamine on the advice of a dermatologist after topical moisturiser failed. The rash completely resolved by day 12 without further management, while other influenza‐like symptoms remained. The loss of taste and smell persisted for ten weeks.

Box 1 **Cutaneous manifestations of COVID‐19 in a 48‐year‐old woman**

**Figure:**
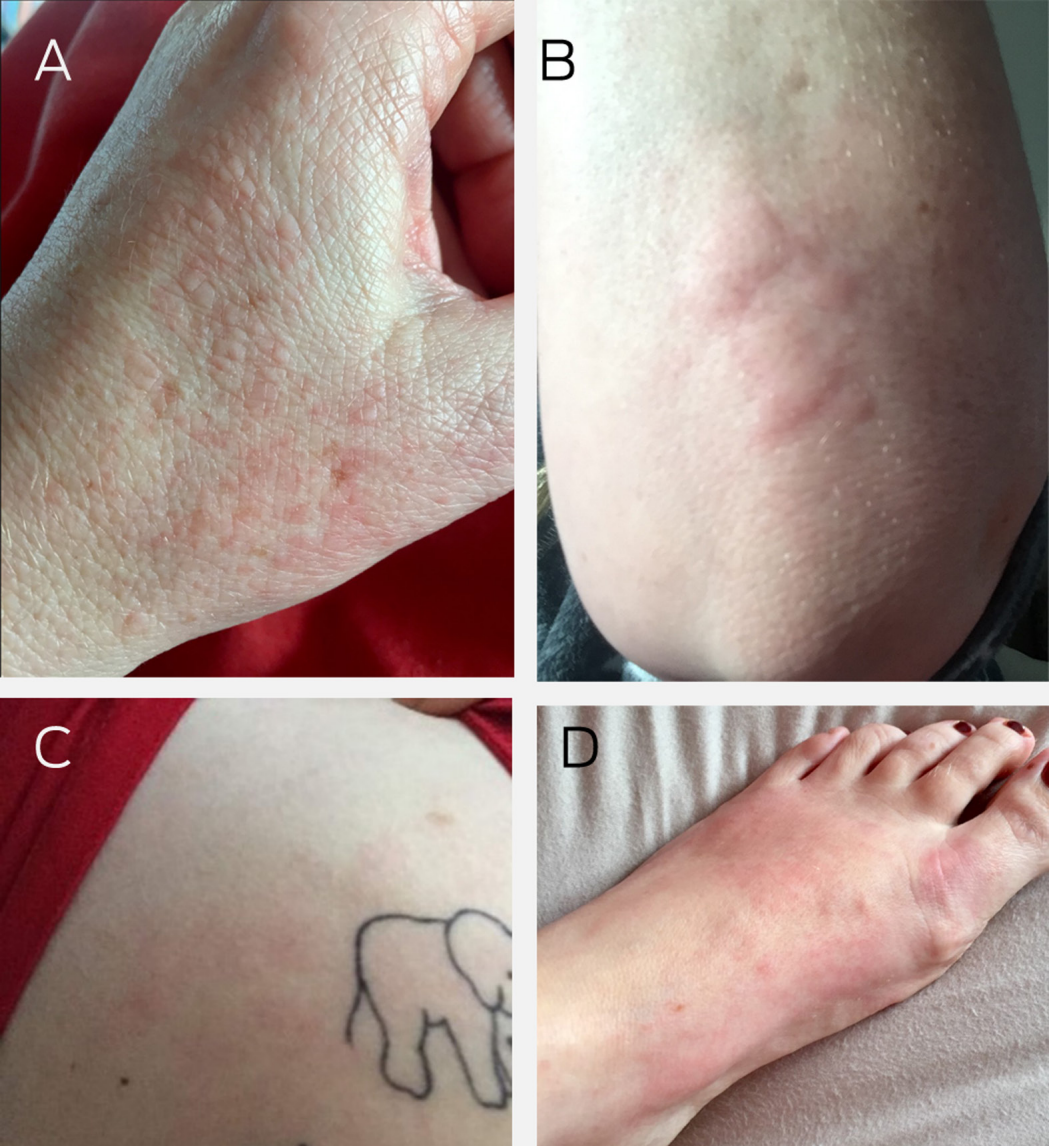
**A:** Maculopapular erythematous eruptions on the patient’s hand. **B:** Wheal and flare pattern of urticaria on her elbow. **C:** Macular erythema and urticarial eruption on her chest. **D:** Macular coalescing erythema on dorsum of her foot with perniosis involvement of toes in areas.

## Discussion

The dermatological manifestations of COVID‐19 were first reported in April 2020.^1^ A study of 1099 hospitalised patients in China found that cutaneous manifestations were only observed in two (0.2%) patients.^1^ Subsequently, a systematic review of 46 articles found that the most commonly reported skin finding was chilblain‐like lesions (402, 40.2%), followed by maculopapular lesions (227, 22.7%), urticarial lesions (89, 8.9%), vesicular lesions (64, 6.4%), livedoid and necrotic lesions (28, 2.8%), and other or nondescript rashes and skin lesions (192, 19.8%).^2^ Other less frequent dermatological manifestations include oral mucosal signs, erythema multiforme‐like lesions, papulosquamous eruptions, dengue‐like rashes, and dry gangrene.^3^ The reported prevalence of cutaneous manifestations of COVID‐19 remains highly variable (Box 2).

Box 2 **COVID‐19‐related rash appearance, localisation, disease association, histopathological findings and management^10^**

**Table:** 

Rash type	Prevalence*	Appearance and localisation	Association with COVID‐19: timing and severity	Histopathology	Management
Perniosis (chilblain)‐like acral lesions (COVID toes)	40.2%	Asymmetric erythematous violaceous or purpuric macules on fingers, elbows, toes, and the lateral aspect of the feet	Late presentation; 2–8‐week resolution time; associated with mild disease	Epidermal necrotic keratinocytes, dermal oedema, perivascular and perieccrine sweat gland lymphocytic inflammation, and vascular changes (endotheliitis and microthrombi)	Tend to self‐resolve; a wait‐and‐see strategy is usually recommended
Exanthematous/morbilliform/ maculopapular rash	22.7%	Pruritic generalised erythematous truncal rash with macules or papules, and sometimes purpura	Present throughout infection; often associated with more severe infection	Vascular damage, perivascular lymphocytic infiltrate, and dense neutrophilic infiltrates	Topical corticosteroids are generally sufficient, but systemic corticosteroids may be appropriate in more severe and widespread presentations
Urticarial rash	8.9%	Transient pruritic welts most often found on the trunk	Appears along with other COVID‐19 symptoms; often associated with more severe disease	Mild lichenoid and vacuolar interface dermatitis with occasional necrotic keratinocytes; associated with mild spongiosis, dyskeratotic basal keratinocytes, and superficial perivascular lymphocytic infiltrates	Antihistamine therapy
Vesicular (varicella‐like) eruptions	6.4%	Vesicular or pustular, varicella‐like eruption on the trunk; may be pruritic	Found early in disease; most commonly associated with mild to moderate infection	Acantholysis, dyskeratosis, unilocular intraepidermal vesicles in a suprabasal location, epidermal necrosis, and endotheliitis in the dermal vessels	Tend to self‐resolve; a wait‐and‐see approach is usually recommended
Livedo reticularis‐like/fixed livedo racemosa/retiform purpura/necrotic vascular lesions	2.8%	Non‐blanching, purple, mottled lace‐like eruption with blood leakage, and necrotic‐vascular lesions; found on the trunk and lower limbs	Usually later onset; associated with severe disease (10% mortality)	Pauci‐inflammatory microthrombotic vasculopathy, with minimal interferon response, and complement‐mediated microvascular injury	Livedo reticularis/racemosa‐like lesions also have a wait‐and‐see strategy due to the absence of effective therapeutic options; purpuric lesions are usually successfully managed with topical corticosteroids; necrotic ulcerative lesions and widespread presentations may be treated with systemic corticosteroids

* Prevalence obtained from a systematic review of 46 articles, including 998 patients with COVID‐19‐related skin manifestations.^2^

The timing of COVID‐19‐related skin manifestations has been linked to the course of the disease: vesicular eruptions early in the disease (15% before other symptoms); and pseudo‐chilblain lesions late in the disease (59% after other symptoms).^4^ Studies are now investigating the possible link between skin manifestations and COVID‐19 illness severity (Box 2). Several critically unwell patients with COVID‐19 have been reported with vasculopathic presentations, including lower limb ischaemia and regions with necrotic or livedoid lesions.^4^ However, chilblain‐like lesions often present in less severe disease. There have also been reports of cutaneous manifestations as the only symptom of COVID‐19 in some patients (1.7%).^5^

Given the association between skin rashes and a positive COVID‐19 swab test result, it is important that clinicians recognise this early clinical feature of COVID‐19.^6^ While following local, up‐to‐date guidelines is necessary regarding COVID‐19 testing, in the case of patients presenting with new chilblain‐like lesions of unclear cause, polymerase chain reaction testing for SARS‐CoV‐2 within 7 days of the onset of lesions may be warranted.^7^ If lesions persist for more than a month, IgM and IgG antibody testing may be more appropriate.^7^

Most cutaneous manifestations of COVID‐19 are self‐resolving. When treatment is appropriate, medium or high potency topical corticosteroids, oral antihistamines or systemic corticosteroids are sufficient for symptomatic relief. Importantly, systemic corticosteroids are not recommended during the acute disease phase, as this may prolong the duration of viral shedding.^8^ Ciclosporin 5 mg/kg/day and intravenous immunoglobulin treatments have been used in severe cases.^9^

Currently, there are no clear management guidelines for COVID‐19‐related skin conditions. A 2021 review recommended the following therapeutic management:^10^

▪ Confluent erythematous/maculopapular/morbilliform eruption management varies according to clinical severity. Topical corticosteroids are often sufficient, with potential use of systemic corticosteroids in more severe presentations.
▪ Papulovesicular exanthems usually self‐resolve in a short time frame. A wait‐and‐see approach is recommended.
▪ Chilblain‐like acral lesions have no widely accepted therapeutic options. These tend to self‐resolve, allowing a wait‐and‐see approach.
▪ Livedo reticularis/racemosa‐like lesions have no known effective treatment options, leaving a wait‐and‐see strategy.
▪ Purpuric lesions are usually successfully managed with topical corticosteroids.
▪ Necrotic ulcerative lesions and widespread presentations may require treatment with systemic corticosteroids.
▪ Urticarial lesions are usually managed with oral antihistamines.

Biopsies are generally only required in severe reactions to exclude a differential diagnosis. Histopathological patterns are summarised in Box 2. However, not all COVID‐19‐related cutaneous manifestations can be classified into these groups.

The use of coinciding drug therapy is a potential confounding factor. Many anti‐COVID‐19 therapies are yet to undergo adequate evaluation in randomised controlled trials. Several agents are currently in clinical trials or have received accelerated approvals. This may be a concern as agents such as hydroxychloroquine, remdesivir, tocilizumab and steroids have known similar cutaneous side effects to SARS‐CoV‐2 infection. A summary of adverse cutaneous events related to the most frequently used drugs in managing COVID‐19 is provided by Martinez‐Lopez and colleagues.^11^

To assist with differentiating viral from therapy‐associated skin reactions, clinicians should take a detailed patient history of relevant drug exposure, as well as any previous history of adverse cutaneous reactions or hypersensitivity.^12^ Serological analysis of lymphocytosis, neutrophilia, eosinophilia, and histamine, tryptase and drug levels, in addition to a histopathological examination for eosinophilia and inflammation, may help determine the cause.^12^ A drug re‐challenge test may also be helpful. The early diagnosis of a drug‐associated cutaneous eruption will allow the clinician to identify the culprit drug and determine if continuing is appropriate.

## Lessons from practice

- Patients with COVID‐19 may present with unusual skin manifestations, including urticarial rashes, vesicular lesions, and chilblains on fingers or toes.
- For patients presenting with new chilblain lesions of unclear cause, polymerase chain reaction testing for SARS‐CoV‐2 within 7 days of the onset of lesions may be warranted. Testing for IgM and IgG antibodies should be considered if lesions persist.
- Most cutaneous manifestations of COVID‐19 are self‐resolving. Where treatment is appropriate, medium or high potency topical corticosteroids, oral antihistamines or systemic corticosteroids are usually sufficient for symptomatic relief.
- Coinciding drug therapy reactions are a possible confounding factor for cutaneous manifestations of COVID‐19.

## Competing interests

No relevant disclosures.

## Provenance

Not commissioned; externally peer reviewed.
